# Osteoregenerative efficacy of a novel synthetic, resorbable Ca/P/S-based bone graft substitute in intra- and peri-articular fractures: a brief medical image-based report

**DOI:** 10.1186/s13018-022-03385-x

**Published:** 2022-11-19

**Authors:** Tai-Hua Yang, Yi-Chuan Chou, Chien-Ping Ju, Jiin-Huey Chern Lin

**Affiliations:** 1 Department of Biomedical Engineering, National Cheng Kung University, 1 University Road, Tainan, 701 Taiwan; 2 Department of Orthopedic Surgery, National Cheng Kung University Hospital, College of Medicine, National Cheng Kung University, Tainan, Taiwan; 3 Department of Materials Science and Engineering, National Cheng-Kung University, Tainan, Taiwan; 4 Center for Biomaterials Research, National Cheng-Kung University, Tainan, Taiwan; 5 Medical Device Innovation Center, National Cheng Kung University, Tainan, Taiwan

**Keywords:** Osteoregneration, Intra-articular fracture, Peri-articular fracture, Inorganic bone substitute

## Abstract

**Background:**

When a fracture goes into or around a joint, it usually damages the cartilage at the ends of bones and other joint tissue. As a result, the affected joints are prone to traumatic arthritis, leading to stiffness. Repairing bone damage, maintaining joint integrity, and avoiding subchondral and metaphyseal defects caused by comminuted fractures is often a great challenge for orthopedic surgeons. Tissue engineering of synthetic bone substitutes has proven beneficial to the attachment and proliferation of bone cells, promoting the formation of mature tissues with sufficient mechanical strength and has become a promising alternative to autograft methods. The purpose of this study is to retrospectively evaluate the clinical outcome and efficacy of a novel synthetic, highly biocompatible, and fully resorbable Ca/P/S-based bone substitute based on medical image findings.

**Materials and methods:**

A synthetic, inorganic and highly porous Ca/P/S-based bone-substituting material (Ezechbone® Granule, CBS-400) has been developed by National Cheng-Kung University. We collected fourteen cases of complex intra- and peri-articular fractures with Ezechbone® Granule bone grafting between 2019/11 and 2021/11. We studied the evidence of bone healing by reviewing, interpreting and analyzing the medical image recordings.

**Results:**

In the present study, CBS-400 was observed to quickly integrate into surrounding bone within three weeks after grafting during the initial callus formation of the early stage of repair. All of these cases healed entirely within three months. In addition, the patient may return to daily life function after 3.5 months of follow-up and rehabilitation treatment.

**Conclusions:**

Ezechbone® Granule CBS-400 was proved capable of promoting bone healing and early rehabilitation to prevent soft tissue adhesions and joint contractures. Moreover, it has a high potential for avoiding ectopic bone formation or abnormal synostosis.

***Trial registration*:**

The Institutional Review Board at National Cheng Kung University Hospital (NCKUH) approved the study protocol (A-ER-109-031, 3-13-2020).

## Background

Intra- or peri-articular fractures occur when bones break into or around a joint. These injuries often damage the cartilage at the ends of bones and other joint tissues. Because fractures in and around the joint tend to damage the cartilage, the affected joint is prone to traumatic arthritis leading to stiffness. The epidemiology of comminuted intra- and peri-articular metaphyseal injuries is often difficult to determine due to differences in target study populations and geographic locations [1, 2]. The principal goal of treatment is primarily to repair bone damage, maintain the joint's integrity and help prevent further joint problems. Subchondral and metaphyseal bone defects caused by comminuted fractures are often a great challenge for orthopedic surgeons. Since the mechanism of support and cartilage maintenance of the articular surface is disrupted, if the damage is not repaired properly, it can lead to sequelae such as nonunion or malunion [3, 4]. Moreover, without proper repair of the bone and joint damage, movement of the injured joint is often avoided to improve stability at the fracture site, thus creating a risk of long-term joint stiffness. Based on the complex morphology of the fracture, surgery is performed for reconstruction that preserves the integrity of the articular surface but often leaves a gap or cavity underneath, resulting in a lack of stability at the fracture site.

Globally, it estimates that there are nearly 2.2 million transplants per year. In the United States alone, more than 500,000 bone grafting surgeries are performed each year, such as in trauma, tumor, spine surgery and revision arthroplasty. Bone graft is the second most-commonly used transplant tissue, next to blood [5–8]. In general, a bone graft may be defined as an implanted material that promotes bone healing with various mechanisms, including osteoinduction (bone morphogenetic proteins, etc.) that is the process by which mesenchymal stem cells (MSCs) at and around the host site are recruited to differentiate into chondroblasts and osteoblasts, osteoconduction (scaffold, etc.) that is the process by which an ordered, spatial three-dimensional ingrowth of capillaries, perivascular tissue, and MSCs takes place from the host site along the implanted graft, and osteogenesis (osteoprogenitor cells, etc.) that is the synthesis of new bone by cells derived from either the graft or the host [9]. Bone graft may help maintain joint stability, integrity and repair periarticular fractures with significant subchondral defects. A bone graft with adequate osteoconduction and/or osteoinduction and strength is preferred for use to fill the space of a bony defect [9]. In addition to providing a mechanical scaffold to help support articular surfaces and maintain alignment, bone graft can also provide structural support for osteocytes during the healing of osteoregeneration, which is a complex cascade of physiological processes of bone formation, found in general fracture healing and involved in continuous remodeling throughout adulthood [10].

Bone grafting is an alternative for addressing bone disease problems and is considered a surgical intervention to facilitate bone healing. Bone grafts can be derived from living donors, post-mortem donors or artificial materials [11–13] and may be categorized into two different types, i.e., biological or synthetic. Synthetic graft materials may further be classified into two groups, osteoinductive material (bone morphogenetic proteins, etc.) and osteoconductive material (scaffold, etc.) [3, 14–16].

Due to its various advantageous features, autologous bone has long been recognized as the gold standard of graft material for bone regeneration [10, 17]. However, its clinical drawbacks, including limited availability and donor site-induced complications and morbidity, limit its use [18–21].Among the recently emerged tissue-engineered new biomaterials to solve these problems, artificial synthetic bone substitutes with different functions have been developed. Studies have proved that these synthetic bone substitutes are beneficial to the attachment and proliferation of bone cells, promoting the formation of mature tissues with sufficient mechanical strength. Tissue engineering of synthetic bone substitutes has proven to be a promising alternative to autograft methods [18, 19, 22–27].

Synthetic bone substitutes may be metallic, ceramic or polymeric. Resorbable bone substitutes typically comprise collagen, hydroxyapatite, tricalcium phosphate, calcium sulfate, or a combination of such minerals in appropriate proportions [10]. Compared with autografts and allografts, artificial bone substitutes have several advantages such as high biocompatibility, absorbability, unlimited supply, ease of sterilization and storage, avoiding the transmission of disease, easy access, and cost-effectiveness [8, 19, 28, 29]. However, most of the currently available bone substitute materials have certain potential disadvantages, such as lack of biocompatibility, inconsistent resorbability and material properties, which can hinder the repair and regeneration of bone tissue [16, 30–32].

The purpose of this study is to retrospectively evaluate the clinical outcome and efficacy of a novel synthetic, highly biocompatible and fully resorbable Ca/P/S-based bone substitute based on medical image findings.

## Materials and methods

**Fig. 1:**
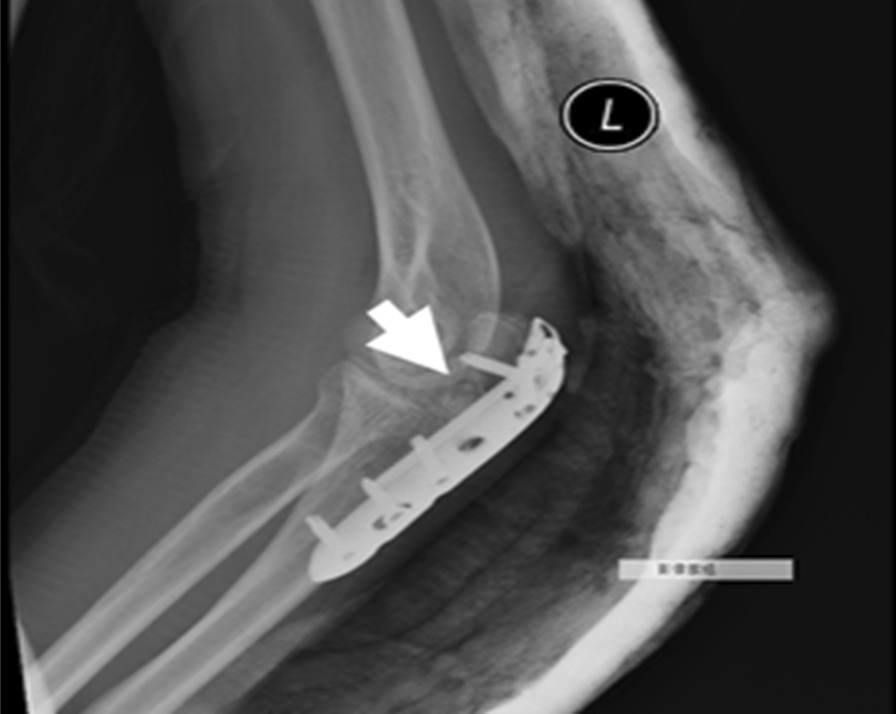
The nidus-like appearance (white arrow) in the X-ray indicates a primary fusion of the bone substitute and its close contact with the surrounding bone

**Fig. 2:**
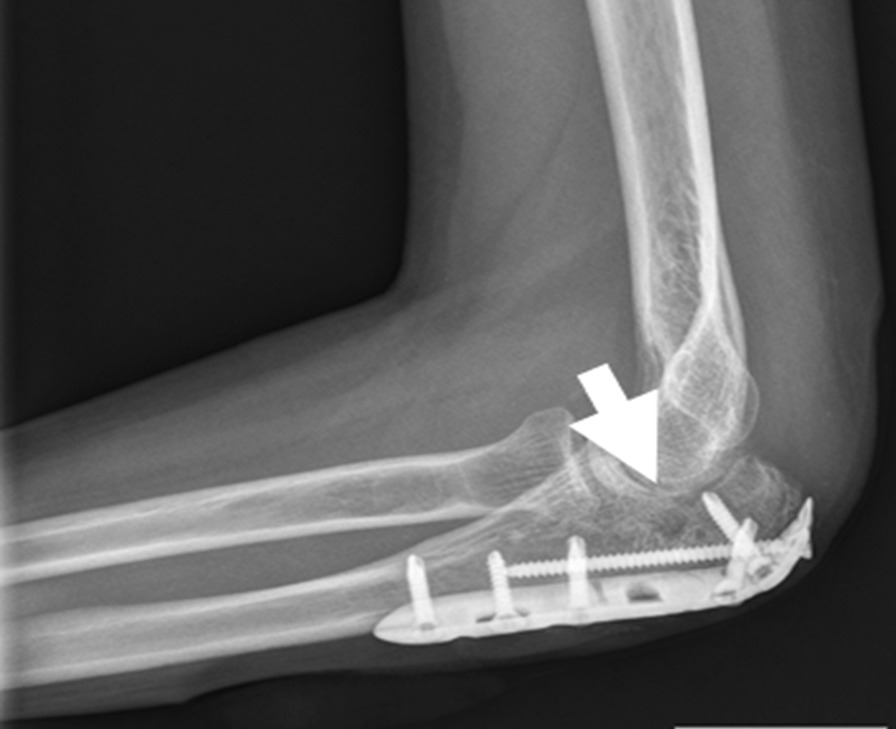
The X-ray reveals a shrinkage of the nidus-like lesion (white arrow) accompanied with initial bone callus formation

**Fig. 3:**
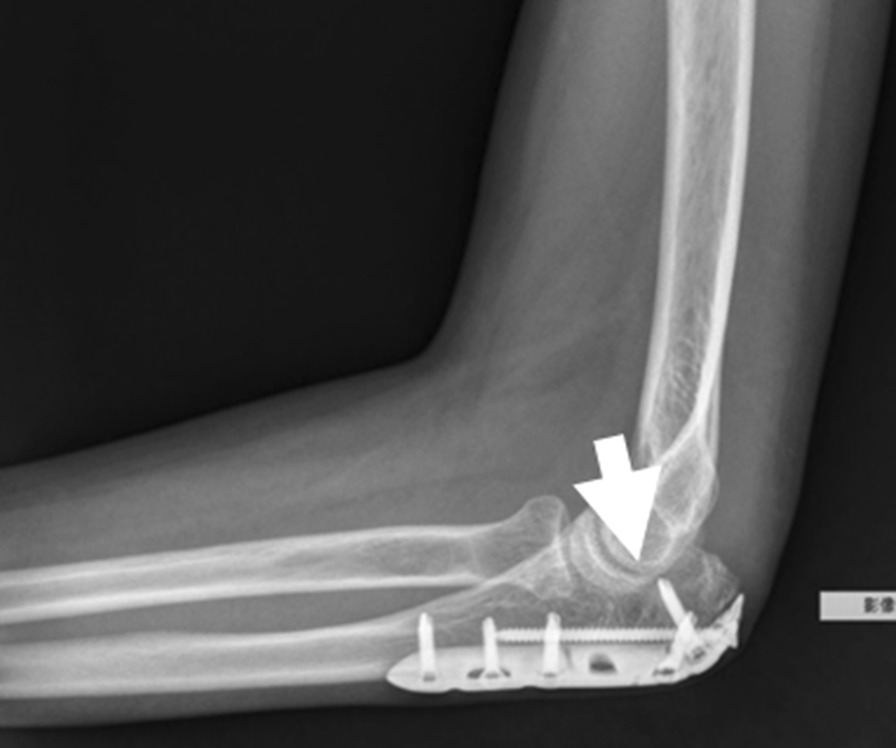
The X-ray-revealed fill-full of the nidus-like lesion (white arrow) in bone graft area demonstrates well healing of the bone

A synthetic, inorganic and highly porous Ca/P/S-based bone-substituting material (Ezechbone® Granule, CBS-400) has been developed by a National Cheng-Kung University (NCKU)/ Joy Medical Devices (JMD) joint research project [29]. Taiwan Food and Drug Administration has approved and granted a product license to the material (Approval No. 003889). Because this is a retrospective study and the study did not adversely affect the rights and welfare of patients, the Institutional Review Board at National Cheng Kung University Hospital (NCKUH) approved the study protocol (A-ER-109-031, 3-13-2020) and agreed to waive the patient's informed consent for data publication. CBS-400 is mainly comprised of hydroxyapatite (HA) and calcium sulfate dihydrate (CSD) with a delicate Ca/P/S atomic ratio of 54.6/39.2/6.2. CBS-400 has demonstrated its excellent biocompatibility from a variety of biocompatibility tests such as cytotoxicity, intradermal reactivity and skin sensitization tests. Animal models also show that the implanted granules are always in intimate contact with the surrounding newly-formed bone. Furthermore, the resorption and formation of new cancellous bone proceed at substantially same pace. In a recent study, the entire process of bone regeneration of CBS-400 revealed a rapid increase in the proportion of new cancellous bone to over 40% at 4 weeks after implantation, followed by a bone remodeling process toward normal cancellous bone. As much as 85% of the bone substitute had been resorbed about 12 weeks after implantation [29, 33]. According to the operation notes from the medical records, we collected fourteen cases of complex intra- and peri-articular fractures with Ezechbone® Granule bone grafting between 2019/11 and 2021/11. We studied the evidence of bone healing by reviewing, interpreting and analyzing the medical image recordings. All imaging images were reviewed, discussed and interpreted by an orthopedic surgeon with 15 + years of clinical experience in the field along with a diagnostic radiologist with 20 + years of experience. As indicated in X-ray, the initial bone graft area had turned into a nidus-like appearance (Fig. 1) several days following grafting, indicating a primary fusion. The shrinkage of the nidus-like lesion (Fig. 2) suggested the initial bone callus formation. The fill-full of the nidus-like lesion (Fig. 3) indicated that the bone healed well. The time required for each different stage was recorded.

## Results

**Fig. 4:**
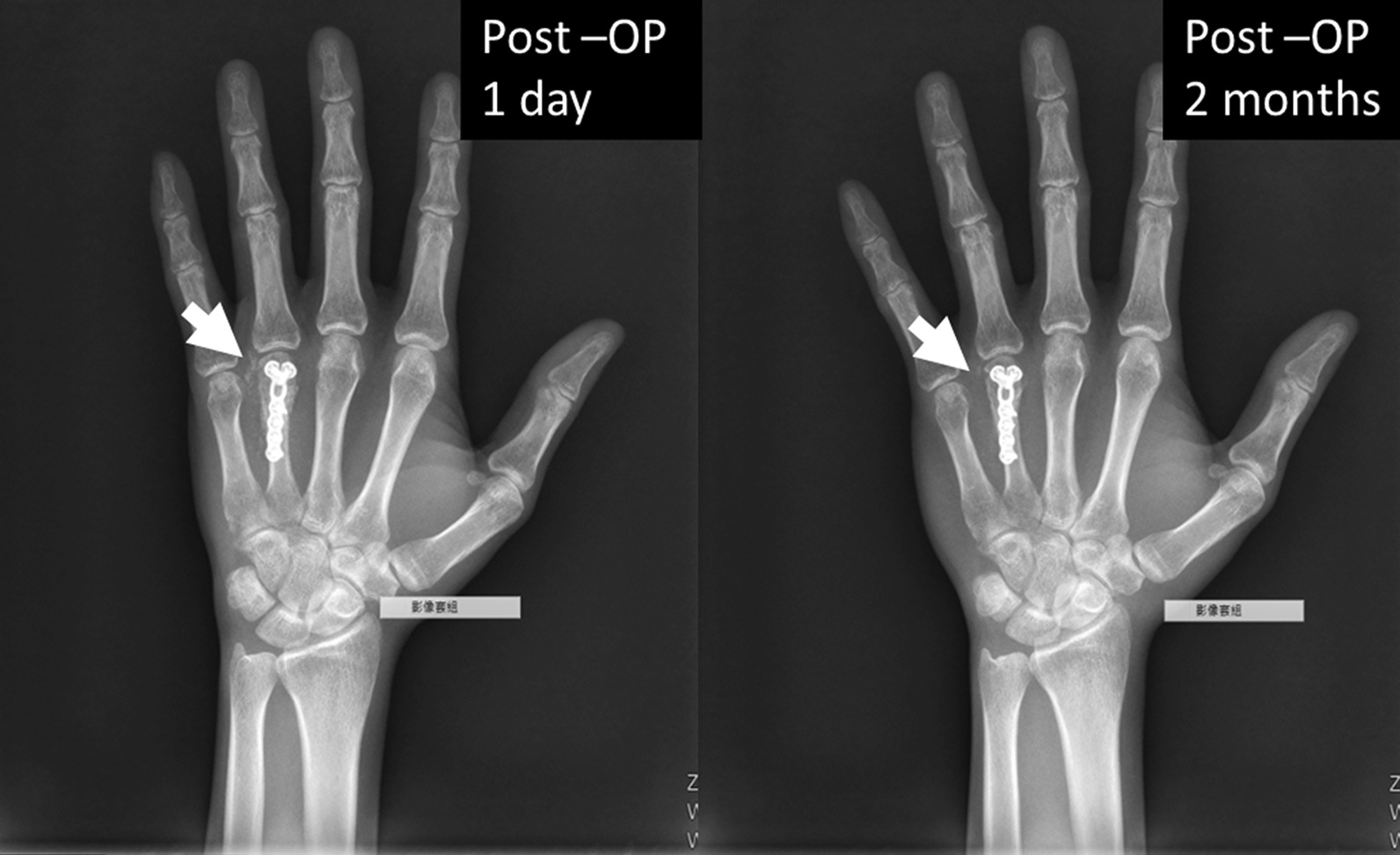
The X-ray shows that the bone substitute material was scattered in the soft tissue around the bone graft area and around the adjacent joint due to the treatment process (left). These implant residues were entirely absorbed (white arrow) within two months after implantation (right)

From November 2019 to November 2021, we followed 14 cases (Table 1), including 11 males and 3 females, with an average of 34.1 ± 9.8 y/o. The fracture types applying bone graft substitutes included 6 peri-articular and 8 intra-articular fractures. The surgical sites were located in two olecranons, two in the distal radius, two at the base of the proximal phalanx, four in the scaphoid, one in the radial head, and three in the metacarpal bones individually. According to medical image interpretation of X-rays, the time to nidus-like formation after bone grafting was 2.3 ± 0.5 weeks; the time to initial callus formation was 4.7 ± 1.0 weeks; and the time to good healing was 10.3 ± 1.3 weeks. In four of the 14 cases, the bone substitute material was scattered in the soft tissue around the bone graft area and around the adjacent joint due to the treatment process (Fig. 4). These implant residues were entirely absorbed within 1.6 ± 0.5 months, for these four cases, and there was no ectopic exostoses formation or synostosis with limited joint mobility. The initial ROM rehabilitation program started on average at 3.0 ± 0.9 weeks. In addition, active joint range of motion and function was restored in all 14 followed-up cases on average at 3.5 months.

**Table 1:** Demographic data and clinical image characteristics

case number	Gender	Age (y/o)	Fracture type	Fracture site	Nidus-like formation (weeks)	Initial callus formation (weeks)	Healing well (weeks)	Initial start to rehabilitation program (weeks)
1	F	34	Intra-articular	Olecraon	2	6	12	3
2	M	26	Intra-articular	Scaphoid	2	6	12	4
3	M	34	Peri-articular	Metacarpal neck	3	5	10	2
4	M	31	Intra-articular	Scaphoid	2	4	8	4
5	M	38	Peri-articular	Metacarpal base	3	6	10	2
6	M	23	Intra-articular	Radial head	2	4	10	6
7	F	55	Intra-articular	Metacarpal base	3	5	10	2
8	F	20	Peri-articular	Proximal phalanx	2	4	10	2
9	M	44	Peri-articular	Proximal phalanx	2	4	8	2
10	M	31	Intra-articular	Scaphoid	2	4	12	4
11	M	23	Peri-articular	Distal radius	3	5	10	3
12	M	44	Intra-articular	Scaphoid	2	3	10	2
13	M	46	Peri-articular	Distal radius	2	4	10	4
14	M	28	Intra-articular	Olecranon	2	6	12	4

## Discussion

It is generally recognized that, in clinical orthopedics, the treatment of complicated fracture, bone defects, delay- or non-union is a challenging and difficult issue. Since Ollier first described the role of bone graft on the procedure for bone healing in 1861, bone grafting has become one major trend clinically. According to the differences among the variety of artificial bone materials, bone graft substitutes can be divided into growth factor-based, cell-based, ceramic-based, and polymer-based materials. The present study is to evaluate the clinical osteoregenerative efficacy of a novel synthetic, highly biocompatible and fully resorbable Ca/P/S-based bone graft substitute based on medical image findings to learn the effectiveness of this material in clinical application, specifically in intra- and peri-articular fractures.

Open reduction and internal fixation usually involves a surgical incision to expose the fracture and using plates and screws to correct/repair the fracture. The fractured bone fragments are screwed back together, allowing direct bone-to-bone healing. Traditionally speaking, bone healing is divided into three stages, including the inflammation stage, repair stage and remodeling stage [34, 35]. The inflammatory stage begins when an injury occurs, while fracture-induced bleeding can form a hematoma or blood clot formation. Local cell death occurs due to tissue damage and chemotactic signaling mechanisms are initiated to clear these cell deaths. At the same time, the blood clot organizes into a network of proteins, and granulation tissue forms between the fragments leading to vascularization of the hematoma [36]. At this stage the transparency of the fracture may increase on radiographs due to bone resorption [34, 37]. This period normally takes about 1–2 weeks. During the following 2–3 weeks, the tissue repair phase begins, where progenitor cells within the granulation tissue proliferate and differentiate into fibroblasts and chondroblasts, producing an extracellular organic matrix of fibrous tissue and cartilage, wherein osteoblasts deposit woven bone [38–40]. At this stage new living cells of bone, cartilage, and fibrous tissue appear at the fracture site, resulting in the formation of rubbery tissue called "fracture callus" or "soft callus." The subsequent calcium deposits in the callus can then begin to be faintly visible on radiographs 2–3 weeks after injury [41] and this phase usually lasts 4–16 weeks. During this phase shear forces can still damage the newly formed callus, while axial traction and pressure promote matrix formation [34, 42]. Finally, bone remodeling occurs when the fractured callus is replaced by solid tissue bone (or called “hard callus”), restoring its typical cortical structure according to Wolff's law related to the load distribution [43]. The overall healing process is ongoing and can last from months to years. The remodeling process is faster in children than in adults that may compensate for malunion to some extent [44, 45].

The bone substitute used in the present study (Ezechbone® Granule CBS-400) was observed to quickly integrate into surrounding bone within three weeks after grafting, while the initial callus formation of calcified deposits could be found within six weeks, indicating the early stage of repair. To the end of the follow-up, all of these cases healed entirely within three months. In addition, the CBS-400-derived earlier callus formation in the early stage of repair made it possible for us to shorten the start time of rehabilitation from 4 to 6 weeks after surgery to within 3 weeks. As a result, the patient may return to daily life function after 3.5 months of follow-up and rehabilitation treatment.

**Fig. 5:**
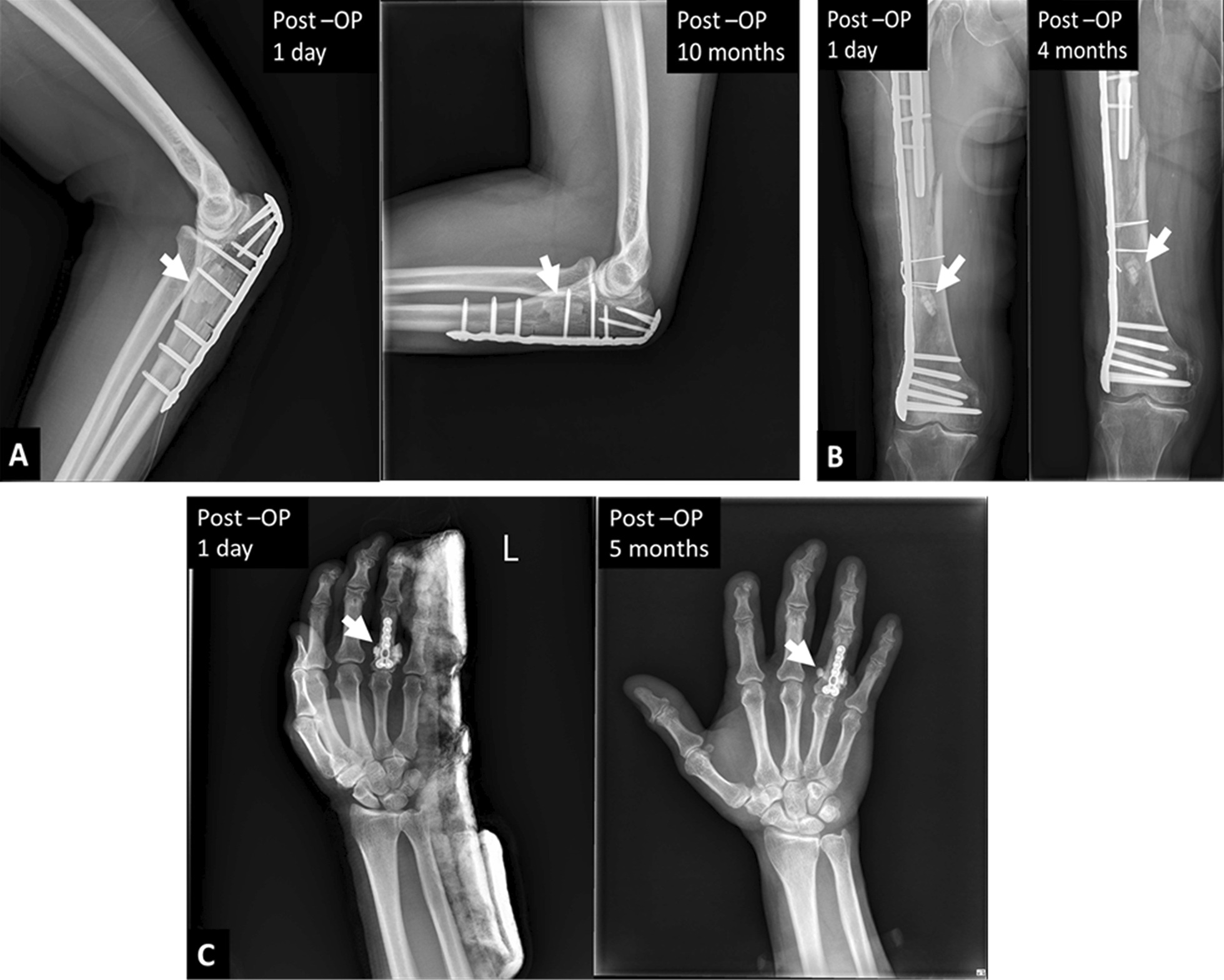
Typical examples demonstrate poor resorbability (**A**, **B**) and ectopic bone formation or abnormal synostosis (**C**) (white arrows) often observed in many commercial brands of synthetic bone graft

An ideal bone graft substitute should present such material properties as biocompatibility, resorbability, vascularity and angiogenesis, durability, osteogenesis and osteoconduction/ osteoinduction. However, in vitro and in vivo studies indicated that most of the artificial bone products in the current market, such as calcium phosphate and recombinant human bone morphogenetic protein, are poorly absorbed and uncertain in biocompatibility, even induce ectopic cartilage and bone formation or abnormal synostosis (Fig. 5A–C) [46–51]. Ezechbone® Granule CBS-400 is highly porous in structure and comprised majorly of Ca-PO_4_ and Ca-SO_4_. It is completely synthetic without biohazards of animal origin, providing excellent biocompatibility and a matched resorption rate to new bone formation. Based on a rabbit animal study [29], the trabecular bone in the implanted region appeared much thicker than that of the non-implanted region, and bone remodeling after 8–12 weeks of implantation of CBS-400 was substantially complete. Microscopic pictures revealed good resorption and integration of the implant with surrounding bone tissues without fibrous formation or inflammatory reaction.

## Conclusions

The primary goal of successful bone augmentation is to provide adequate intensity for early active and passive range-of-motion exercises to prevent soft tissue adhesions and joint contractures. From the present preliminary clinical results, the present Ca/P/S-based bone-substituting material (Ezechbone® Granule CBS-400) provides capability of promoting bone healing. Moreover, it has a high potential for avoiding abnormal ectopic bone formation or synostosis. Collection of more cases and further in-depth study are invited to reassure its efficacy.

## Data Availability

All data generated or analysed during this study are included in this published article.

## Abbreviation

MSCs: Mesenchymal stem cells
NCKUH: National Cheng Kung University Hospital
NCKU: National Cheng-Kung University
JMD: Joy medical devices
HA: Hydroxyapatite
CSD: Calcium sulfate dihydrate
